# Functional Regulation of PPARs through Post-Translational Modifications

**DOI:** 10.3390/ijms19061738

**Published:** 2018-06-12

**Authors:** Reinhard Brunmeir, Feng Xu

**Affiliations:** 1 Lee Kong Chian School of Medicine, Nanyang Technological University, 11 Mandalay Road, Singapore 308232, Singapore; Reinhard.Brunmeir@gmail.com; 2 Institute of Molecular and Cell Biology, Agency for Science, Technology and Research (A*STAR), 61 Biopolis Drive, Singapore 138673, Singapore; 3 Department of Biochemistry, Yong Loo Lin School of Medicine, National University of Singapore, 8 Medical Drive, Singapore 117596, Singapore

**Keywords:** nuclear receptors, PPARα, PPARγ, PPARβ/δ, post-translational modifications

## Abstract

Peroxisome proliferator-activated receptors (PPARs) belong to the nuclear receptor superfamily and they are essential regulators of cell differentiation, tissue development, and energy metabolism. Given their central roles in sensing the cellular metabolic state and controlling metabolic homeostasis, PPARs became important targets of drug development for the management of metabolic disorders. The function of PPARs is mainly regulated through ligand binding, which induces structural changes, further affecting the interactions with co-activators or co-repressors to stimulate or inhibit their functions. In addition, PPAR functions are also regulated by various Post-translational modifications (PTMs). These PTMs include phosphorylation, SUMOylation, ubiquitination, acetylation, and *O*-GlcNAcylation, which are found at numerous modification sites. The addition of these PTMs has a wide spectrum of consequences on protein stability, transactivation function, and co-factor interaction. Moreover, certain PTMs in PPAR proteins have been associated with the status of metabolic diseases. In this review, we summarize the PTMs found on the three PPAR isoforms PPARα, PPARβ/δ, and PPARγ, and their corresponding modifying enzymes. We also discuss the functional roles of these PTMs in regulating metabolic homeostasis and provide a perspective for future research in this intriguing field.

## 1. Introduction

**Figure 1:**
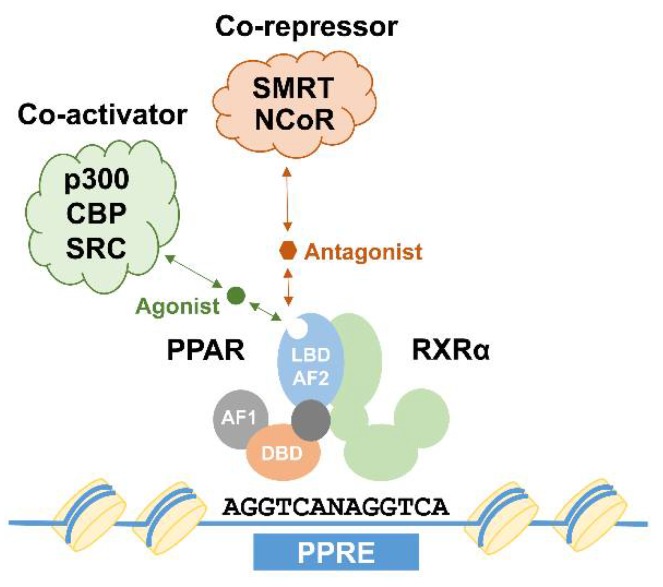
Transcriptional regulation by peroxisome proliferator-activated receptor (PPAR) proteins. PPARs form dimers with Retinoic acid receptor α (RXRα) proteins and subsequently bind to a DNA sequence known as peroxisome proliferator response elements (PPRE). Binding of agonists (green circle) or antagonists (red hexagon) lead to structural changes, enhancing co-activator (such as p300, CREB-binding protein (CBP), and Steroid receptor coactivator 1 (SRC1)) or co-repressor (such as Nuclear receptor corepressor (NCoR) and the Silencing mediator of retinoid and thyroid hormone receptor (SMART)) binding. AF1: activation function 1 domain; DBD: DNA-binding domain; LBD-AF2: ligand binding and activation function 2 domain.

Nuclear receptors (NRs) are Transcription factors (TFs) capable of ligand binding, which modulates their activities to regulate gene expression. In this way, NRs directly process external signals to adapt relevant gene expression programs. Peroxisome proliferator-activated receptors (PPARs) are representative members of this large superfamily of NRs, which consist of three closely related isotypes: PPARα (NR1C1, encoded by the *Ppara* gene), PPARβ/δ (NR1C2, encoded by the *Ppard* gene), and PPARγ (NR1C3, encoded by the *Pparg* gene). The overall structure of PPAR proteins (and other NRs) is highly conserved and consists of six functional domains, A to F. The N-terminal portion of PPARs (domains A/B) is termed as the Activation-function 1 (AF-1) domain responsible for transcriptional activation. It provides constitutive activation function independent of ligand binding. The AF-1 domain is followed by a DNA-binding domain (DBD, domain C), containing two zinc-finger motifs involved in DNA recognition and protein-protein interaction. Finally, a more flexible hinge domain (domain D) is succeeded by the C-terminal Ligand-binding domain (LBD, domains E/F), which contains not only the ligand-binding pocket, but also regions important for dimerization, and the AF-2 domain. Ligand binding is thought to induce structural changes of the AF-2 domain, allowing the recruitment of co-activator proteins important for transcriptional activation, thereby serving as a switch to activate PPARs. To exert their biological functions, PPAR proteins form heterodimeric complexes with Retinoic acid receptor α (RXRα), another member of the NR family, through their dimerization domain. Binding to RXRα is a prerequisite for PPARs to bind to DNA, which usually occurs at regions known as PPAR response elements (PPREs) containing the conserved DNA sequence motif AGGTCANAGGTCA. PPAR:RXR heterodimers not bound to a ligand are thought to act as repressors through association with co-repressor complexes such as Nuclear receptor corepressor (NCoR) and the Silencing mediator of retinoid and thyroid hormone receptor (SMART). In contrast, ligand binding mediates the recruitment of co-activator complexes containing p300, CREB-binding protein (CBP), or Steroid receptor coactivator 1 (SRC1) to the heterodimers, leading to subsequent transcriptional activation of their target genes (Figure 1).

A broad variety of natural compounds has been found to bind and activate PPAR proteins. Those natural ligands include fatty acids and their derivatives, coming either from external sources (diet) or arising as products of internal metabolic processes (de novo lipogenesis, lipolysis, etc.). Thus, via their sensitivity to intracellular levels of metabolites, PPARs act as sensors of the cellular metabolic states. Moreover, they have the ability to adjust gene regulatory networks according to fluctuating metabolic demands. Therefore, it is not surprising that PPARs have a central role in various cellular pathways linked to the energy homeostasis including glucose metabolism, lipid uptake and storage, insulin sensitivity, mitochondrial biogenesis, and thermogenesis. With the rise of metabolic disorders, commonly subsumed under the term “metabolic syndrome”, over the last decades, PPAR proteins have emerged as interesting therapeutic targets to counter pathological conditions such as obesity, Type 2 diabetes (T2D), insulin resistance, Nonalcoholic fatty liver disease (NAFLD), Nonalcoholic steatosis (NASH), dyslipidema, and hypertension [1,2]. Numerous synthetic ligands targeting one, two, or all three PPARs have been developed and have entered various stages of (pre-)clinical trials, with several gaining admission. Currently, fibrates (synthetic PPARα agonists) are used to treat dyslipidemia, whereas the class of antidiabetic Thiazolidinediones (TZDs) targeting PPARγ had been widely prescribed for the management of T2D but are now partially withdrawn from clinical use due to their side effects [3,4,5].

The three different isoforms of PPAR have overlapping, but distinct roles, owing to their expression profiles in different tissues, sensitivities to agonists, and regulation of target genes (Reviewed in: [6]). PPARα is highly expressed in kidney, liver, Brown adipose tissue (BAT), heart, and skeletal muscle, the tissues with high capacities for Fatty acid oxidation (FAO). Accordingly, its main role seems to be the control of energy dissipation through the regulation of lipid metabolism in response to nutritional changes (such as fasting and feeding). PPARβ/δ shows a relatively broader expression pattern, with enriched levels in tissues associated with fatty acid metabolism, such as the gastrointestinal tract, heart, kidney, skeletal muscle, fat, and skin. Its physiological role in energy homeostasis is complex, as it not only controls plasma lipid levels through FAO in several tissues, but also modulates glucose handling in muscle and liver. The third member of the PPAR family, PPARγ, exists in two distinct protein forms: the shorter PPARγ1—lacking its first 30 amino acids due to alternative promoter usage—is expressed in a broad variety of cells including immune and brain cells, whereas the full length isoform PPARγ2 is highly abundant in BAT and White adipose tissue (WAT). PPARγ2 is considered the master regulator of adipocyte differentiation and stimulates energy storage by controlling fatty acid uptake and lipogenesis [7].

Many proteins undergo Post-translational modifications (PTMs), i.e., the covalent attachment of chemical groups to certain amino acid residues, at some points of their life-cycle. Those PTMs range from small entities such as methyl-, acetyl-, or phospho-groups to sizeable polypeptides such as ubiquitin chains with a size of several kDa. Their addition can have a wide spectrum of consequences on the chemical properties of targeted proteins, which further modulate protein functions. As expected, PTMs are important regulators of virtually every aspect of protein biology, including protein stability, cellular localization, enzyme function, and co-factor interaction. Several excellent recent reviews have covered various aspects of PPAR biology, including their roles in metabolic diseases [8], energy homeostasis [6], and as drug targets [9]. This review aims to give an overview of the current status of research on PTMs found in PPARα, PPARβ/δ, and PPARγ, and their functional roles. 

## 2. Post-Translational Modifications of PPARα

### 2.1. Phosphorylation

**Figure 2:**
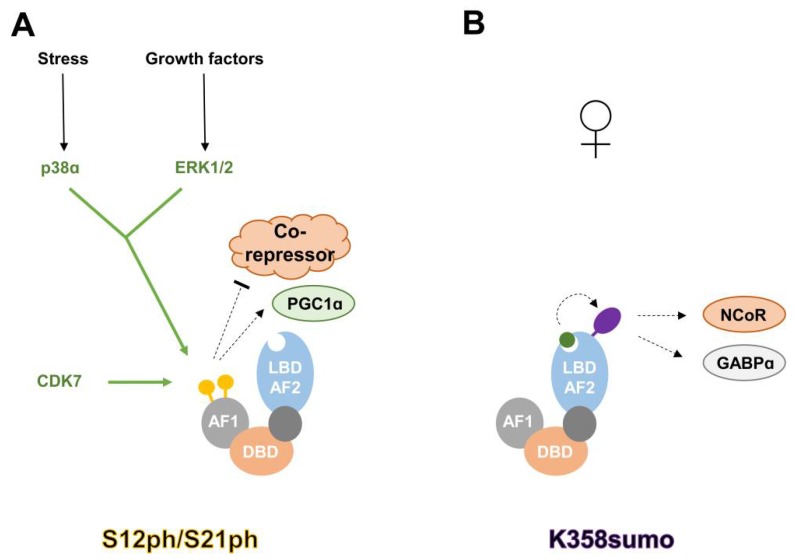
Regulatory mechanisms of S12ph/S21ph and K358sumo in PPARα. (**A**) Phosphorylation of serine 12 and 21 enhances PPARα activity, most likely via reduced co-repressor and/or increased Peroxisome proliferator-activated receptor gamma coactivator 1-alpha (PGC1α) recruitment. Both residues are targeted by Mitogen-activated protein kinase (MAPK) downstream kinases p38 and Extracellular signal–regulated kinase 1/2 (ERK1/2), as well as Cyclin-dependent kinase (CDK) 7. (**B**) Upon ligand binding, PPARα gets SUMOylated at K358 in female liver cells, leading to increased binding of NCoR and GA-binding protein α (GABPα), and silencing of androgen steroid genes. AF1: activation function 1 domain; DBD: DNA-binding domain; LBD-AF2: ligand binding and activation function 2 domain; enzymes depositing post-translational modifications (PTMs) are colored in green; green arrows indicate deposition of PTMs; green circle: PPARα-ligand; yellow circle: phosphorylated serine; purple oval: SUMOylated lysine; black arrow: activation; dotted arrow: increased interaction/stimulation; dotted T symbol: decreased interaction.

It was reported as early as 1996 [10] that PPARα is a phosphoprotein. Its phosphorylation was shown to increase upon treatment with different stimuli such as insulin [10] and ciprofibrate, a PPARα agonist [11]. Specific serine residues in PPARα have emerged as important phosphorylation sites: serine 12 and 21, which are both targeted by either Mitogen-activated protein kinases (MAPKs) [12,13] or Cyclin-dependent kinase (CDK) 7 [14]. Functionally, phosphorylation of S12/S21 (S12ph/S21ph) correlates with increased transactivation of PPARα in hepatocytes and cardiac myocytes, potentially via decreased co-reperessor interaction (NCoR) or increased interaction with certain co-activators (Peroxisome proliferator-activated receptor gamma coactivator 1-alpha (PGC1α)). Lower S12ph/S21ph (together with decreased PPARγ phosphorylation, see below) is observed in Xeroderma pigmentosum group D (XPD) patients, which carry a mutation in the CDK7-containing Transcription factor II H (TFIIH) complex, and might partially explain their complex metabolic phenotypes, including reduced adipose mass and increased energy expenditure [14]. Another important phosphorylation event regulating PPARα function, S73ph, is mediated by Glycogen synthase kinase β (GSKβ), and leads to the degradation of PPARα [15]. Interestingly, in a mouse model of Gilbert’s Syndrome, it was shown that the protective effect against hepatic steatosis might be mediated by increased PPARα protein levels and reduced S73ph [16]. A recent publication also reported increased S12ph in peripheral blood mononucleated cells of Gilbert’s Syndrome patients [17]. The regulatory mechanism of S12ph/S21ph in PPARα is illustrated in Figure 2A.

### 2.2. SUMOylation

SUMO (Small Ubiquitin-like MOdifier) polypeptides are roughly 12 kDa in size, which can be covalently attached to lysine residues via an enzymatic machinery analogous to that for protein ubiquitination. Its addition can have a wide range of effects on protein function [18]. Two lysine residues of PPARα have been reported to be subjected to this modification: K185 and K358 [19,20]. While SUMOylation of both residues increases the repressive ability of PPARα through enhanced co-repressor recruitment (NCoR, or GA-binding protein (GABP)), their regulation by PPARα agonists is marked different: K185sumo is blocked by the PPARα ligand GW7647, whereas agonist mediated conformational change of the LBD seems a prerequisite for efficient K358 SUMOylation. Functionally, K358 SUMOylation plays an interesting role in the establishment of sexual dimorphism of liver cells. The modification only occurs in female livers, where it helps to repress genes involved in the production of androgen steroids. The regulatory mechanism of K358sumo in PPARα is illustrated in Figure 2B.

### 2.3. Ubiquitination

There is a body of work showing that PPAR protein levels are regulated by the ubiquitin proteasome system [21]. Early findings implicated the E3 ligase function of Mouse double minute 2 homolog (MDM2) in the regulation of PPARα protein stability [22]. More recently, the addition of a single ubiquitin (mono-ubiquitination) has emerged as another way to regulate PPARα function in cardiomyocytes. Rodriguez et al. [23] found that the muscle-specific ubiquitin ligase Muscle-specific RING finger protein 1 (MuRF1) can modify PPARα, leading to the decreased activity of PPARα due to its export from the nucleus to the cytoplasm. Three lysine residues (K292, K310, and K358) located around a newly identified nuclear export signal in the LBD (aa300-308) were identified as putative mediators of this effect. 

## 3. Post-Translational Modifications of PPARγ

### 3.1. Phosphorylation

PPARγ is by far the best studied member of the PPAR family, and phosphorylation of PPARγ has been reported as early as 1996 [24,25], shortly after its discovery as the master regulator of adipogenesis [7]. Numerous reports in quick succession showed that PPARγ gets phosphorylated upon stimulation of the MAPK activated pathway [24,25,26,27,28]. A variety of stimuli such as growth factors (Epidermal growth factor (EGF), Platelet-derived growth factor (PDGF), Transforming growth factor β (TGFβ) and insulin), Prostaglandin F2α (PGF2α), or cellular stress (UV, 12-*O*-tetradecanoyl-13-phorbol acetate (TPA) and anisomycin) were shown to trigger PPARγ phosphorylation through the activation of the downstream Extracellular signal-regulated kinases (ERKs) 1/2 or p38/c-Jun *N*-terminal kinase (JNK). The phosphorylation site was mapped to PPARγ2 serine 112 (corresponding to PPARγ1 S82), located in the AF1 region within a MAPK consensus site [24,28]. The functional role of S112ph was revealed through reporter assays, where the phosphorylation led to decreased transcriptional activity of PPARγ. Mutagenesis experiments further corroborated the notion that S112ph inhibits PPARγ function, as the expression of a nonphosphorylatable S112A led to increased transcriptional activity and enhanced adipogenic potential of fibroblasts [24,26,27,28,29,30,31,32,33]. On the flipside, the same mutation is detrimental for efficient osteoblast differentiation [34,35]. Another publication highlighted the role of the adaptor molecule Docking protein 1 (DOK1) as a modulator of this signaling cascade: DOK1 is induced by High fat diet (HFD) feeding and negatively regulates ERK1/2 mediated S112ph, thereby enhancing PPARγ activity even in a state of active insulin signaling [36]. Finally, our understanding of the mechanisms by which S112 gets dephosphorylated is also improved by the identification of Protein phosphatase 5 (PP5) [37], Protein phosphatase Mg^2+/^Mn^2+^ dependent 1B (PPM1B) [38], and Wild-type p53-induced phosphatase 1 (WIP1) [39] as S112 phosphatases and PPARγ activators.

How is the repressive function of S112ph mediated mechanistically? Adams et al. showed that the phosphorylation event does not appear to impact PPARγ protein stability, or reduce its DNA binding activity. Instead, they proposed that S112ph might inhibit the transactivation function of PPARγ via co-repressor recruitment or co-activator release [26]. In another study, S112ph was shown to modulate PPARγ function by reducing ligand binding affinity, which involves the intramolecular communication between the AF1 and the ligand binding domain [30]. Finally, Grimaldi et al. described a mechanism by which S112ph regulates PPARγ-mediated transcription: phosphorylation of S112 enhances the interaction between PPARγ and the circadian clock protein Period circadian regulator 2 (PER2). PPARγ-PER2 interaction was shown to be detrimental to PPARγ recruitment to general adipogenic regulators as well as BAT-specific genes, such as *Ucp1*, *Elovl3*, and *Cidea*. Consequently, knockout of PER2 was found to cause increased BAT gene expression and oxidative capacity in WAT [40].

S112 is not exclusively targeted by the MAPK signaling pathway. Using the same xeroderma pigmentosum model mentioned earlier, Compe et al. [14] observed lower levels of PPARγ S112ph (together with decreased PPARα phosphorylation (see above)), which they attributed to the disruption of the CDK7 containing TFIIH complex. Indeed, they showed that CDK7 phosphorylates S112 in vitro. The authors also found reduced trans-activator function of PPARγ in their xeroderma pigmentosum system, and suggested a model where S112ph by CDK7 activates PPARγ function, in opposition to the repressive S112ph mediated by MAPK signaling. This result has been put into perspective by Helenius et al. [41], who found that MAT1, another THIIH complex member, and CDK7 itself, not only enhanced S112ph, but also inhibited adipocyte differentiation, which is in line with a generally repressive role of S112ph. Finally, another publication added the positive adipogenic regulator CDK9 to the list of S112ph kinases [42].

The physiological importance of S112ph has been highlighted by several lines of evidence: (1) In a (homozygous) S112A knock-in mouse model, Rangwala et al., found that the S112A mutation protects mice from obesity induced insulin resistance [43]; (2) A meta-analysis of Genome-wide association studies (GWAS) confirmed that the occurrence of the S112A allele is correlated with reduced type 2 diabetes risks [44]; and (3) subjects with the rare heterozygous variant P113Q, which renders the neighboring S112 nonphosphorylatable and increases its adipogenic potential [31], causes a range of metabolic symptoms ranging from obesity, type 2 diabetes, insulin resistance, and high fasting insulin levels [31,45]. This indicates that the phenotypic consequences are highly dependent on the genetic background, as well as the nutritional status. Additional studies will be necessary to untangle the complex relationship between genotype, PTM status, environmental cues, and disease risk.

In 2010, Choi et al. [46] revealed another phosphorylation event of PPARγ, S273ph, and since then this modification has attracted considerable interest. Serine 273 was found to be located within the consensus motif of CDK5, and readily get phosphorylated by the activated form of this kinase. Similar to S112, the loss of phosphorylation at S273 had activating effects on PPARγ, but the exact biological consequences were quite distinct: it did not increase the overall adipogenic activity of PPARγ, but upregulated a specific subset of target genes promoting insulin sensitivity. Mechanistically, this was caused by the loss of phosphorylation-dependent recruitment of the co-factor Thyroid hormone receptor associated protein 3 (THRAP3) [47]. Increased S273ph (which was induced by obesity) could be counteracted using PPARγ agonists, which led to improved metabolic profiles in HFD mice and patients with impaired glucose tolerance. Crucially, PPARγ binding compounds inhibiting S273ph with no or very low agonist activities elicited similar effects, without the side effects like weight gain, fluid retention, and bone loss, usually seen with PPARγ activation by full agonists such as TZDs [34,46,48]. Therefore, blocking S273ph seems to be an interesting avenue to treat metabolic disorders and a number of such compounds have been developed recently [47,48,49,50]. It will be intriguing to see their clinical potential in the future. In support of this notion, decreased S273ph was also detected in two genetic knockout models connected to an improved metabolic status in mice [51,52].

In a follow up paper to their work that identified CDK5 as a S273 kinase, Banks et al. generated adipocyte specific CDK5 knockout mice, and to their surprise found that S273ph levels were increased rather than decreased upon the ablation of this kinase [53]. This was explained by enhanced MEK/ERK (Extracellular signal–regulated kinase) signaling (caused by loss of CDK5), as ERK was subsequently identified as another potent S273ph kinase. In line with that notion, MEK inhibitor treatment produced beneficial metabolic effects [53]. In another publication, pharmacological inhibition of CDK5 via roscovitine evoked a somewhat different effect as genetic ablation, decreasing S273ph as well as S112ph, enhancing expression of BAT genes, increasing energy expenditure, and improving metabolic profiles [54]. This demonstrates that although the manipulation of signaling pathways connected to PPARγ phosphorylation is a highly promising approach to ameliorate metabolic disorders, more experimental work is needed to gain a comprehensive understanding of the underlying mechanisms.

**Figure 3:**
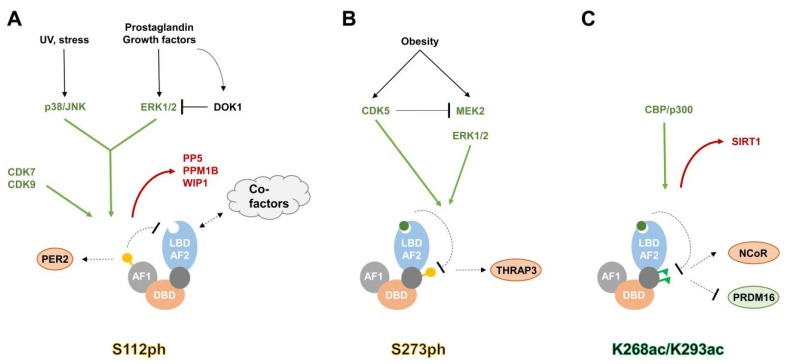
Regulatory mechanisms of selected modifications in PPARγ. (**A**) Activation of the MAPK pathway leads to the phosphorylation of serine 112 by p38/JNK or ERK1/2. S112ph decreases PPARγ activity, either through reducing its ligand binding affinity and co-activator binding, or by increasing Period circadian regulator 2 (PER2) binding, which leads to decreased recruitment to target genes. The adapter molecule Docking protein 1 (DOK1) modulates S112ph levels in response to nutritional inputs. S112ph is also targeted by CDK7 and CDK9. Phosphatases removing S112 phosphorylation from PPARγ are: Protein phosphatase 5 (PP5), Protein phosphatase Mg^2+/^Mn^2+^ dependent 1B (PPM1B), and Wild-type p53-induced phosphatase 1 (WIP1). (**B**) Obesity-induced MAPK signaling leads to serine 273 phosphorylation, which enhances binding of the Thyroid hormone receptor associated protein 3 (THRAP3), and repression of certain PPARγ target genes. Phosphorylation levels are modulated by CDK5, either directly by CDK5-medatied S273 phosphorylation, or indirectly via phosphorylation of Dual specificity mitogen-activated protein kinase kinase 2 (MEK2) and suppression of MAPK signaling. Compounds with or without PPAR agonist activity can be used to block S273ph. (**C**) Acetylation of lysines 268 and 293 has been shown to increase NCoR co-repressor binding, whereas NAD (Nicotinamide adenine dinucleotide)-dependent deacetylase sirtuin-1 (SIRT1)-mediated deacetylation of K293 favours PR domain containing 16 (PRDM16) binding and expression of thermogenic genes. Ligand binding enhances SIRT1-PPARγ interaction and K268/K293 deacetylation. AF1: activation function 1 domain; DBD: DNA-binding domain; LBD-AF2: ligand binding and activation function 2 domain; enzymes depositing PTMs are colored in green, enzymes removing PTMs are shown in red; green circle: PPARγ-ligand; yellow circle: phosphorylated serine; green triangle: acetylated lysine; black arrow: activation; green arrow: PTM deposition; red arrow: PTM removal; black T symbol: inhibition; dotted arrow: increased interaction/stimulation; dotted T symbol: decreased interaction/inhibition.

Another important direction will be the identification and characterization of novel phosphorylation events in PPARγ. S112 and S273 are clearly not the only phosphorylated residues within PPARγ, as Banks et al. identified further phosphorylated sites (S133, T296) by Liquid chromatography-tandem mass spectrometry (LC-MS/MS) [53]. In addition, Choi et al. [55] recently described the phosphorylation of Y78, regulated by SRC proto-oncogene, nonreceptor tyrosine kinase (c-SRC), and Protein-tyrosine phosphatase 1B (PTP-1B), to be important for the regulation of genes involved in cytokine and chemokine expression. The regulatory mechanisms of S112ph and S273ph in PPARγ are illustrated in Figure 3A,B, respectively.

### 3.2. SUMOylation

PPARγ SUMOylation with SUMO1 was first reported in 2004 [56,57,58]. The targeted lysine residue was identified as K107 on PPARγ2, located within a SUMOylation consensus motif (K77 in PPARγ1) [56,57,58]. Through analysis of cells expressing K107R mutant, it was found that the lack of PPARγ K107 SUMOylation correlated with transcriptional activation of PPARγ target genes [56,57,58,59], and enhanced adipogenesis [56]. These studies clearly defined K107 SUMOylation as a repressive mark for PPARγ, although the exact mechanism still remains to be elucidated. One proposed mechanism—the destabilization of PPARγ [58]—is most likely not the only important functional consequence of SUMO ligation. In support of this view, in the macrophage cell system, where PPARγ1 has a role in the repression of inflammatory genes, K77 SUMOylation was found to be important for the anti-inflammatory response triggered by apoptotic cells, possibly through stabilization of the co-repressor NCoR at target genes [60]. This is reminiscent of the effect of another SUMOylation event described earlier: also working in a macrophage cell system, Pascual et al. [61] showed that TZD-mediated SUMO1-modification of K365 (K395 in PPARγ2) is important for the repression of inflammatory response genes via PPARγ binding and stabilization of an NCoR-containing repressive complex. The precise biological roles of both modifications in the anti-inflammatory response, especially potential functional overlaps, remain to be determined. 

A more recent publication reported that PPARγ can also be targeted by the SUMO2 modification and identified K33, K64, K68, and K77 (K63, K94, K98, and K107 in PPARγ2) as target sites, of which the first three sites are located within an inverted SUMOylation consensus motif. SUMOylation at either position was reported to be detrimental to PPARγ trans-activation [62]. 

The enzymatic machinery mediating PPARγ SUMOylation and de-SUMOylation has been identified earlier and consists of Ubiquitin conjugating enzyme 9 (UBC9, E2 ligase) [56,59,61], Protein inhibitor of activated STAT (PIAS1/PIASxβ, E3 ligase) [57,61,63,64], and SUMO-specific protease 2 (SENP2, protease) [65].

Interestingly, several reports have linked K107sumo to another PTM occurring in close proximity: S112ph. Initial reports showed that S112A, but not S112D phosphor-mimetic mutations, decreased PPARγ2 SUMOylation and transactivation function [56,59], supporting the model of a phospho-SUMOyl switch to regulate PPARγ function [66]. However, there might be additional mechanisms, allowing K107sumo regulation independent of S112ph. This notion is supported by two lines of evidence: (1) In Fibroblast growth factor 21 (FGF21) knockout mice, where PPARγ-dependent gene expression was reduced, increased K107sumo was not accompanied by elevated S112ph (and S273ph) [67]; and (2) Growth differentiation factor 11 (GDF11) treatment, which inhibits adipogenic differentiation and enhances osteoblastogenesis, increased PPARγ SUMOylation, again without concomitant changes of S112ph (and S273ph) [68].

While many details of the exact mechanisms and pathways governing SUMO-mediated PPARγ regulation remain open to future research, work from Mikkonen et al., has highlighted its physiological importance, as they showed that SUMO1 knockout mice exhibited a metabolic phenotype and decreased PPARγ target gene expression [69].

### 3.3. Acetylation

It was first noted in 2010 that PPARγ is a target for lysine acetylation [70], but only in 2012 another report gave a more detailed insight into its biological function [71]. Qiang and coworkers [71] identified five acetylated lysine residues at position K98, K107, K218, K268, and K293, of which two (K268ac and K293ac) could by blocked by administration of the TZD rosiglitazone, or by activation of the NAD (Nicotinamide adenine dinucleotide)-dependent deacetylase sirtuin-1 (SIRT1) deacetylase. It turned out that deacetylation of both residues, as seen in SIRT1 gain-of-function models, had beneficial metabolic effects, leading to browning of WAT and insulin sensitization. Mechanistically, this was achieved by modulation of co-factor recruitment. In detail, deacetylation of K293 favored the binding of the brown adipogenic activator PR domain containing 16 (PRDM16), whereas acetylation of K268 and K293 enhanced interaction with the co-repressor NCoR. Another mass spectrometric approach led to the identification of a total of nine putative acetylation sites on PPARγ1 (including the lysine residues corresponding to K218 and K268 on PPARγ2), of which K154 and K155 (K184 and K185 in PPARγ2) were further characterized [72]. K154/K155A and K154/K155Q mutants both showed severely diminished lipogenic potential compared to the WT protein. The regulatory mechanism of K268/K293ac in PPARγ is illustrated in Figure 3C.

### 3.4. Ubiquitination

Recently, two publications identified Seven in absentia homolog 2 (SIAH2) and Makorin RING finger protein 1 (MKRN1) as PPARγ E3 ligases, targeting PPARγ for proteasomal degradation [73,74]. MKRN1 activity was mainly directed towards K184 and K185. This work enhanced earlier work on PPARγ regulation through modulation of its stability (reviewed in [21]). A more unusual function for PPARγ ubiquitination was reported by two other publications: Watanabe et al. [75] and Li et al. [76] showed that the E3 ligases Tripartite motif containing 23 (TRIM23) and Neural precursor cell expressed, developmentally downregulated 4 (NEDD4) confer atypical poly-ubiquitination to PPARγ (non-K48-mediated formation of poly-ubiquitin chains), which leads to reduced proteasomal degradation and stabilization of PPARγ.

### 3.5. O-GlcNAcylation

The addition of the single sugar modification β-*O*-linked *N*-acetylglucosamine (*O*-GlcNAc) to serine and threonine residues has been proposed to act as a nutrient sensor, linking signal transduction and gene expression to the metabolic status. Therefore it is interesting that PPARγ1 has been reported to get modified at T54 (corresponding to T84 of PPARγ2), leading to a decrease of its trans-activator function [77]. 

## 4. Post-Translational Modifications of PPARβ/δ

### SUMOylation

PPARβ/δ is the least studied PPAR family member, and to our knowledge there is only one publication reporting a PTM in it: Koo et al. [78] show that PPARβ/δ SUMOylation at K104 is removed by SENP2, and (together with PPARγ, which is also targeted by SENP2, see above) this promotes the expression of FAO genes in muscle.

**Figure 4:**
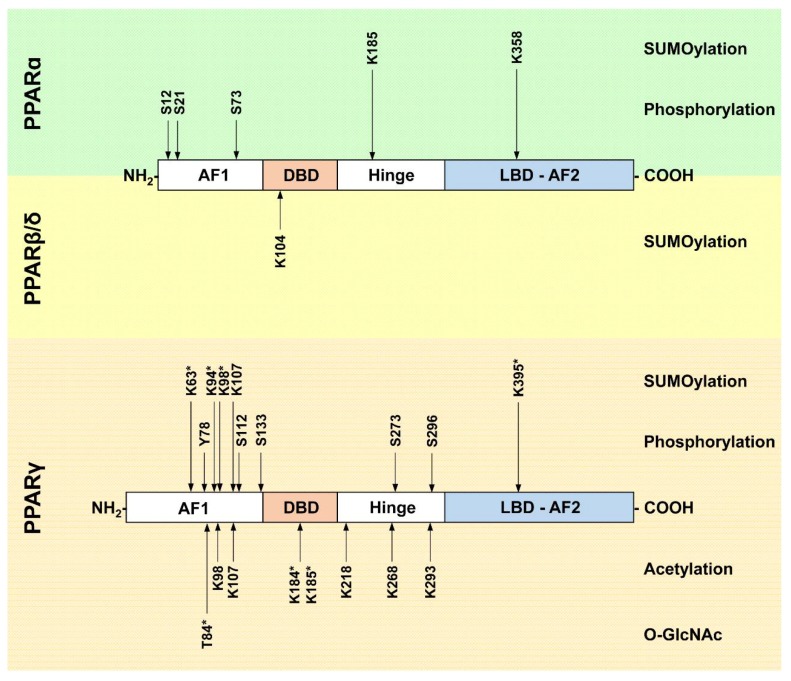
Post-translational modification sites in PPAR proteins. A schematic view of PPARα, PPARβ/δ, and PPARγ proteins and their functional domains is provided. The locations of PTM sites are indicated by arrows and the amino acid positions are given. Note that amino acids positions correspond to the murine proteins. For PPARγ, all amino acid positions refer to the PPARγ2 sequence; modifications which have so far only been described in PPARγ1 are highlighted with an asterisk. Ubiquitination events are not shown. AF1: activation function 1 domain; DBD: DNA-binding domain; Hinge domain; LBD-AF2: ligand binding and activation function 2 domain; K—lysine, S—serine, Y—tyrosine, T—threonine.

The PTMs in PPAR proteins and their corresponding modifying enzymes discussed above are summarized in Figure 4 and Table 1.

## 5. Outlook/Perspective

The last years have seen a wealth of information gathered on the role of PTMs on PPAR proteins. It is evident that PTMs are powerful modulators of PPAR function and we are getting an increasingly clearer picture of its complexity. They influence almost every aspect of PPAR biology, ranging from protein stability, localization, 3D structure, to ligand binding and co-factor interaction. 

PTMs are the results of the action of signaling cascades, and therefore can be seen as representation of the physiological state of a cell. This is strikingly similar to the role of PPAR ligands, metabolites which are representing the metabolic status of a cell. PTMs and ligands can therefore be interpreted as two distinct, but related and partially overlapping, input signals and routes to modulate PPAR activity. It is not surprising that numerous instances of crosstalk between PTMs and agonist/antagonist action have been reported, but substantially more work is needed to dissect this complex network of relationships. 

In the future, the use of high-throughput techniques will be instrumental to tackle questions related to the role of PTMs for target gene binding and genomic localization (via Chromatin immunoprecipitation-sequencing (ChIP-seq) using modification-specific antibodies), or the discovery of additional modifications (such as methylation) via mass-spectrometry based proteomic assays. The latter approach has already led to the identification of a fast growing number of new modification sites [53,71,72]. Due to increasing numbers of modifications, future studies will face the challenge that they will not only have to address their individual roles, but also take into consideration potential crosstalk between modifications. Introducing another layer of complexity is the fact that a growing number of amino acid residues has been shown to get targeted by more than one modification (e.g., PPARγ2 K98 and K107 can get SUMOylated as well as acetylated). This will make it necessary to revisit earlier results and critically re-evaluate some of the previous conclusions. Especially, assays based on the mutation of targeted residues might require careful reanalysis. Finally, it will be interesting to interrogate putative functional links between disease-risk connected Single-nucleotide polymorphisms (SNPs) and their potential effects on PTMs (as has been done for PPARγ2 S112ph and P113Q).

Importantly, some of those findings might lead to new approaches to tackle the prevalent epidemic of metabolic disorders. For example, the discovery that phosphorylation and acetylation events correlate with certain metabolic outcomes lends weight to the suggestions to specifically modulate responsible signaling pathways. A more directed approach, tackling not entire signaling pathways, but specifically blocking the modification of PPAR proteins itself via small molecules, seems to be an even more promising avenue that could decrease off-target/side effects. An example for the latter option is the use of small molecules to inhibit PPARγ2 S273ph [47,48,49,50]. It will be interesting to see if this approach can be successfully translated into the clinics and extended to other PTMs.

In summary, with an improving understanding of PPAR biology in general, and the role of PTMs specifically, PPARs remain promising targets for clinical interventions and will be in the focus of interest for years to come.

## Figures and Tables

**Table 1:** Summary of PPAR modifying enzymes. Enzymes that deposit modifications are highlighted in green, while enzymes removing modifications are shown in red. For PPARγ, amino acid sequence positions refer to PPARγ2. If there is only experimental evidence for modification in PPARγ1, the corresponding amino acid position in PPARγ2 is given and highlighted with an asterisk. Question marks indicate undetermined target sites.

Modification	Enzyme	Target-Site	References
Phosphorylation	ERK1/2	PPARα S12, S21 PPARγ S112, S273, S133	[13,24,26,29,53,68]
	p38-α	PPARα S12, S21	[12]
	CDK7	PPARα S12, S21 PPARγ S112	[14,41]
	GSKβ	PPARα S73	[15]
	JNK	PPARγ S112	[26]
	CDK9	PPARγ S112	[42]
	CDK5	PPARγ S112, S273, S296	[46,53]
	MEK2	PPARγ S133	[53]
	c-SRC	PPARγ Y78	[55]
	PP5	PPARγ S112	[37]
	PPM1B	PPARγ S112	[38]
	WIP1	PPARγ S112	[39]
	PTB-1B	PPARγ Y78	[55]
Acetylation	CBP	PPARγ K98, K107, K218, K268, K293	[71]
	p300	PPARγ K?	[70]
	SIRT1	PPARγ K184/185 *, K268, K293	[70,71,72]
SUMOylation	PIAS1/PIASxβ	PPARα K358 PPARγ K107, K395 *	[20,57,61,63,64]
	PIASy	PPARα K185	[19]
	UBC9	PPARα K185 PPARγ K107, K395 *	[19,56,59,61]
	SENP2	PPARγ K107 PPARβ/δ K104	[65,78]
Ubiquitination	MKRN1	PPARγ K184/185	[74]
	SIAH2	PPARγ K?	[73]
	NEDD4	PPARγ K?	[76]
	TRIM23	PPARγ K?	[75]
	MDM2	PPARα K?	[22]
	MuRF	PPARα K?	[23]
O-GlcNAcylation	OGT	PPARγ T84 *	[77]

## Abbreviations

PPAR	Peroxisome Proliferator Activated Receptor
NR	Nuclear receptor
TF	Transcription factor
AF-1	Activation-function 1
DBD	DNA-binding domain
LBD	Ligand-binding domain
RXRα	Retinoic acid receptor α
PPRE	PPAR response element
NCoR	Nuclear receptor corepressor
SMART	Silencing mediator of retinoid and thyroid hormone receptor
CBP	CREB-binding protein
SRC1	Steroid receptor coactivator 1
T2D	Type 2 diabetes
NAFLD	Nonalcoholic fatty liver disease
NASH	Nonalcoholic steatosis
TZD	Thiazolidinedione
BAT	Brown adipose tissue
FAO	Fatty acid oxidation
WAT	White adipose tissue
PTM	Post-translational modification
MAPK	Mitogen-activated protein kinase
CDK	Cyclin-dependent kinase
PGC1α	Peroxisome proliferator-activated receptor gamma coactivator 1-alpha
XPD	Xeroderma pigmentosum group D
TFIIH	Transcription factor II H
GSKβ	Glycogen synthase kinase β
SUMO	Small ubiquitin-like modifier
GABP	GA-binding protein
MuRF1	Muscle-specific RING finger protein 1
EGF	Epidermal growth factor
PDGF	Platelet-derived growth factor
TGFβ	Transforming growth factor β
TPA	12-*O*-tetradecanoyl-13-phorbol acetate
PGF2α	Prostaglandin F2α
ERK	Extracellular signal–regulated kinase
MEK2	Dual specificity mitogen-activated protein kinase kinase 2
JNK	c-Jun *N*-terminal kinase
DOK1	Docking protein 1
HFD	High fat diet
PP5	Protein phosphatase 5
PPM1B	Protein phosphatase Mg^2+^/Mn^2+^ dependent 1B
WIP1	Wild-type p53-induced phosphatase 1
PER2	Period circadian regulator 2
GWAS	Genome-wide association study
THRAP3	Thyroid hormone receptor associated protein 3
LC-MS/MS	Liquid chromatography-tandem mass spectrometry
c-SRC	SRC proto-oncogene, non-receptor tyrosine kinase
PTP-1B	Protein-tyrosine phosphatase 1B
UBC9	Ubiquitin conjugating enzyme 9
PIAS	Protein inhibitor of activated STAT
SENP2	SUMO-specific protease 2
FGF21	Fibroblast growth factor 21
GDF11	Growth differentiation factor 11
SIRT1	NAD-dependent deacetylase sirtuin-1
PRDM16	PR domain containing 16
SIAH2	Seven in absentia homolog 2
MKRN1	Makorin RING finger protein 1
TRIM23	Tripartite motif containing 23
NEDD4	Neural precursor cell expressed, developmentally down-regulated 4
*O*-GlcNAc	β-*O*-linked *N*-acetylglucosamine
ChIP-seq	Chromatin immunoprecipitation-sequencing
SNP	Single-nucleotide polymorphism
